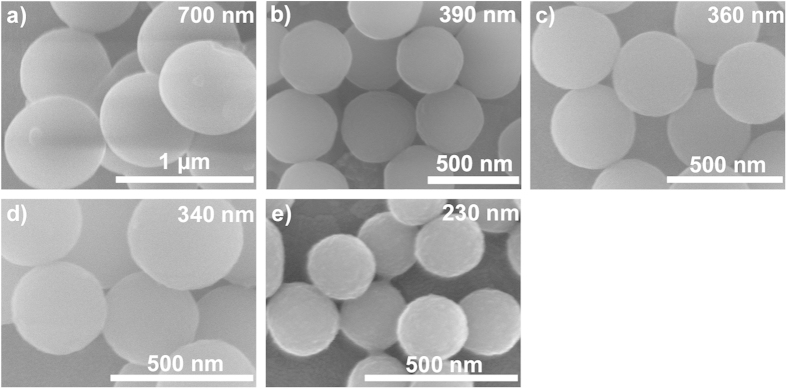# Corrigendum: Selecting water-alcohol mixed solvent for synthesis of polydopamine nano-spheres using solubility parameter

**DOI:** 10.1038/srep13473

**Published:** 2015-09-01

**Authors:** Xiaoli Jiang, Yinling Wang, Maoguo Li

Scientific Reports 4 Article number: 607010.1038/srep06070; published online 06142014; updated 09012015

This Article contains errors in Figs 2 and 3. Figure 2d was inadvertently duplicated in Fig. 2e; Fig. 3c was inadvertently duplicated in Fig. 3b. The correct Figs 2 and 3 appear below as Figs 1 and 2 respectively.

## Figures and Tables

**Figure 1:**
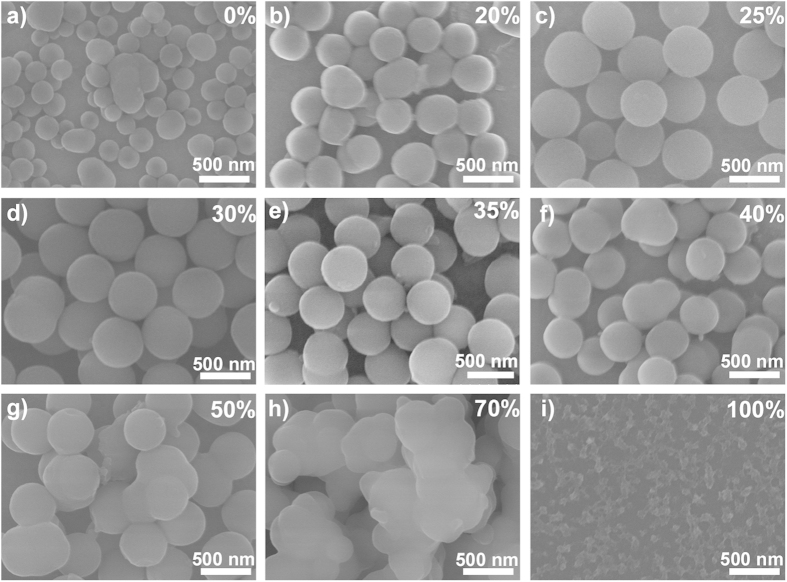


**Figure 2:**